# ﻿Discovery of *Whittieriahengduanensis* sp. nov. (Ophioglossaceae) from Southwest China demonstrates a unique intercontinental disjunct pattern in plants between the Himalaya and the Americas

**DOI:** 10.3897/phytokeys.249.135379

**Published:** 2024-11-12

**Authors:** Zhen-Long Liang, Li-Bing Zhang

**Affiliations:** 1 Key Laboratory of Mountain Ecological Restoration and Bioresource Utilization, Chengdu Institute of Biology, Chinese Academy of Sciences, P.O. Box 416, Chengdu, Sichuan 610041, China Chengdu Institute of Biology, Chinese Academy of Sciences Chengdu China; 2 University of Chinese Academy of Sciences, Beijing 100049, China University of Chinese Academy of Sciences Beijing China; 3 Missouri Botanical Garden, 4344 Shaw Blvd., St. Louis, MO 63110, USA Missouri Botanical Garden St. Louis United States of America

**Keywords:** Adder’s tongues, fern biogeography, intercontinental disjunctions

## Abstract

A new fern species, *Whittieriahengduanensis* (Ophioglossaceae), from Sichuan, Xizang, and Yunnan, Southwest China (eastern Himalaya), is described and illustrated. This species is similar to *W.engelmannii* in the Americas in having a cylindrical rhizome and complex-reticulate venation. In addition, both species grow in open habitat on basic soil. However, the two species are distinguishable in root number per rhizome and the number of the larger areolae per trophophore. Our molecular study also supports that they are sister to each other but divergent at the molecular level. The discovery of *W.hengduanensis* shows that the genus is intercontinentally disjunct between the Himalaya and the Americas, a unique pattern not having been documented in the literature.

## ﻿Introduction

Ophioglossaceae or Adder’s tongue ferns are known for their extremely simplified morphological characteristics (Clausen 1938; Wagner 1990; Zhang et al. 2020; Zhang and Zhang 2022) and the highest known chromosome numbers among known organisms (Ghatak 1977). Following the most recent phylogenetic analysis based on Sanger DNA sequence data, plastome sequences, and analyses of 34 morphological/ecological traits, this family was divided into four subfamilies and 15 genera (Zhang and Zhang 2022). Among the four subfamilies, Ophioglossoideae are the most difficult taxonomically because of their extremely simple morphology. Zhang and Zhang (2022) recognized seven genera in Ophioglossoideae: *Cheiroglossa* C. Presl, *Goswamia* Li Bing Zhang & Liang Zhang, *Haukia* Li Bing Zhang & Liang Zhang, *Ophioderma* (Blume) Endl., *Ophioglossum* L., *Rhizoglossum* C. Presl, and *Whittieria* Li Bing Zhang & Liang Zhang. Based on plastome and mitogenome data, Kuo et al. (2024) resolved the morphologically well-defined South African *Rhizoglossum* as sister to *Whittieria* and found the overall relationships to be consistent to those found by Zhang and Zhang (2022), albeit with lower support values, especially for the monophyly of *Ophioglossum* s.l. (31% maximum likelihood bootstrap value). Kuo et al.’s (2024) results are suggestive that the recognition of the seven genera in the subfamily is well founded.

Among the seven genera in Ophioglossoideae, *Whittieria* was believed to be monospecific and endemic to North to Central Americas. This genus is well defined morphologically, ecologically, and physiologically in Ophioglossoideae by having complex-reticulate venation (large areoles of the trophophore subdivided into smaller areoles; Wagner and Wagner 1994), growing in basic soil, and having relatively shorter spore germination time (70 days vs. 90–100 days of species in *Ophioglossum* s.s.; Whittier 1981).

During a field trip in western Sichuan and southeastern Xizang in 2021, we collected some materials of a species of Ophioglossoideae from several localities. This species has quite clear complex-reticulate venation, similar to the American *Whittieriaengelmannii*, but about 8000 km away from the latter species’s distribution in air distance. We then conducted detailed morphological and phylogenetic analyses and found that this represents a new species of *Whittieria*.

## ﻿Materials and methods

### ﻿Morphology study

Plant materials were collected from the field trips in Sichuan and Xizang in 2021. The collected specimens were compared with other herbarium specimens deposited at CDBI, and PE, as well as digital images from online sources such as CVH (https://www.cvh.ac.cn/), PPBC (https://ppbc.iplant.cn/), and POWO (https://powo.science.kew.org/).

A preliminary morphological study showed that the newly collected material had complex-reticulate venation with large areolae including several small areolae, which resembled the American genus, *Whittieria*.

### ﻿DNA sequencing and phylogenetic study

To resolve the relationships of the newly collected materials from western Sichuan, we included 32 samples of Ophioglossoideae representing six out of the seven genera recognized by Zhang and Zhang (2022). The South African *Rhizoglossum* C. Presl was not sampled. One species from each of *Cheiroglossa* and *Ophioderma* was used as outgroups following the most recent phylogenies of the family published by Zhang et al. (2020) and Zhang and Zhang (2022).

Silica gel-dried materials were collected in the field. Total genomic DNA was extracted from silica gel-dried samples using the FOREGENE Plant Genomic DNA Isolation Kit. Three plastid markers were sequenced for the phylogenetic analysis, the *rbcL* gene and two intergenic spacers, *rps4-trnS* and *trnL-F*, were separately amplified using the standard PCR protocol. The lab work, sequence alignments, and phylogenetic analysis followed Zhang et al. (2020) and Zhang and Zhang (2022).

The resulting DNA sequences were deposited in GenBank. The information on the plant materials used in the sequencing along with GenBank accession numbers are listed in the Appendix 1.

## ﻿Results

**Figure 1.**
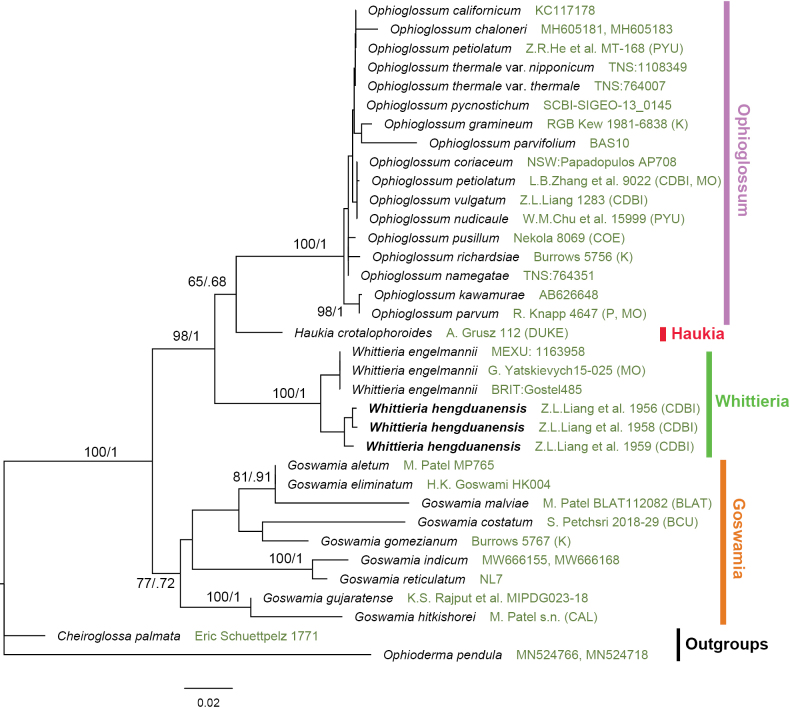
The maximum likelihood tree based on the combined plastid dataset of *rbcL*, *trnL-F*, and *rps4-trnS*. Maximum likelihood bootstrap support (MLBS) and Bayesian inference posterior probability (BIPP) are given above and below the branches, respectively. Voucher information is indicated behind the species name.

Nine sequences of three samples were newly sequenced. The combined dataset of *rbcL*, *trnL-F*, and *rps4-trnS* contained 3,029 bp of which 462 sites were parsimoniously informative. In spite of only three markers and limited taxon sampling used to construct the phylogeny (Fig. 1), all four segregated genera of *Ophioglossum* s.l. recognized by Zhang and Zhang (2022) were recovered as monophyletic except the monospecific *Haukia*, of which only one accession was included. *Goswamia* was resolved as sister to the rest, and *Whittieria* was sister to a clade containing *Ophioglossum* s.s. and *Haukia*. The newly sampled three accessions of the southwestern Chinese (eastern Himalayan) species were resolved as monophyletic and were sister to *W.engelmannii*.

## ﻿Discussion

Consistent with previous findings (Zhang et al. 2020; Zhang and Zhang 2022; Kuo et al. 2024), *Haukia* is resolved as sister to *Ophioglossum* s.s. (Fig. 1). However, *Goswamia* is resolved as sister to the rest of the four genera, which differs from a resolution in which *Goswamia* was found to be sister to *Whittieria* (Zhang et al. 2020; Zhang and Zhang 2022). We consider this conflict as minor because our current resolution of *Goswamia* received low statistical support. This conflict might have been the result of different sampling sizes and characters included.

It is quite striking that the eastern Himalayan species (hereafter referred as *Whittieriahengduanensis*) is resolved as sister to the American endemic *W.engelmannii*. The genus *Whittieria* was believed to contain a single species in America (Zhang and Zhang 2022). With the second species discovered here, this genus now has an intercontinental disjunction between the Himalaya and the Americas. Wen (1999) listed 65 genera of seed plants displaying the eastern Asian-eastern North American disjunctions. Xiang et al. (2015) hypothesized that 31 groups of ferns and lycophytes showed eastern Asian-North American disjunctions. However, none of these taxa are endemic to the Himalaya and have their sister in the Americas (southern North America and Central America for *Whittieria*). Therefore, the intercontinental disjunction pattern in *Whittieria* has not yet been documented in any group of plants at any taxonomic ranks. The divergence time between *Whittieria* and the rest of Ophioglossoideae and that between two species in *Whittieria* are of great biogeographical significance. Where is the ancestral area of *Whittieria* then? How did the two species form today’s disjunct pattern? The discovery of *W.hengduanensis* is surely interesting.

*Whittieriahengduanensis* is indeed similar to *W.engelmannii* in having complex-reticulate venation, which is unique in Ophioglossaceae and in fact defines the genus *Whittieria* (Zhang and Zhang 2022). The morphological differences between the two species are minute (see below) because of the generally simple morphology in the subfamily. Both species grow in basic soil, which is unique in the family, too (Zhang and Zhang 2022). However, the two species are so widely displaced geographically (ca. 8000 km in air distance), with *W.hengduanensis* being in the eastern part of the Himalaya and *W.engelmannii* being endemic to North and Central America (Wagner and Wagner 1994; Wan et al. 2022). In addition, the two species occur in very different elevations with *W.hengduanensis* in elevations between 2500–4000 m with temperate and alpine climates and *W.engelmannii* in those between 200–2200 m with tropical, subtropical, and temperate climates.

For a long time, this species has been confused with *Ophioglossumnudicaule* L.f. They overlap in geographical distribution. However, *O.nudicaule* has no persistent old leaf stalks at the base of the rhizomes and fewer roots per rhizome, and the sporophore base is not slightly attached to the trophophore. Importantly, they have different venation patterns with *W.hengduanensis* with complex-reticulate venation and *O.nudicaule* with common reticulate venation, although Wagner and Wagner (1994) reported complex-reticulate venation for the North American *O.nudicaule*. It is unclear whether the materials from Africa (type locality), Asia, and the Americas of “*O.nudicaule*” represent the same species.

## ﻿Taxonomic treatment

### ﻿ Whittieria hengduanensis

Taxon classificationPlantaeOphioglossalesOphioglossaceae

Z.L.Liang & Li Bing Zhang sp. nov.

0829AA53-9D6B-5C81-ABE9-958450C592DC

urn:lsid:ipni.org:names:77351700-1

Figs 2 , 3

#### Type.

China • Sichuan: Yajiang County, Jiaonibao Village, elev. 2750 m, 30°6'10.93″N, 101°1'49.79″E, in the shrubs in dry and hot river valleys, 15 July 2021, *Z.-L. Liang, L.-S. Jiang & Q. Yu LZL1959* (holotype CDBI!).

**Figure 2.**
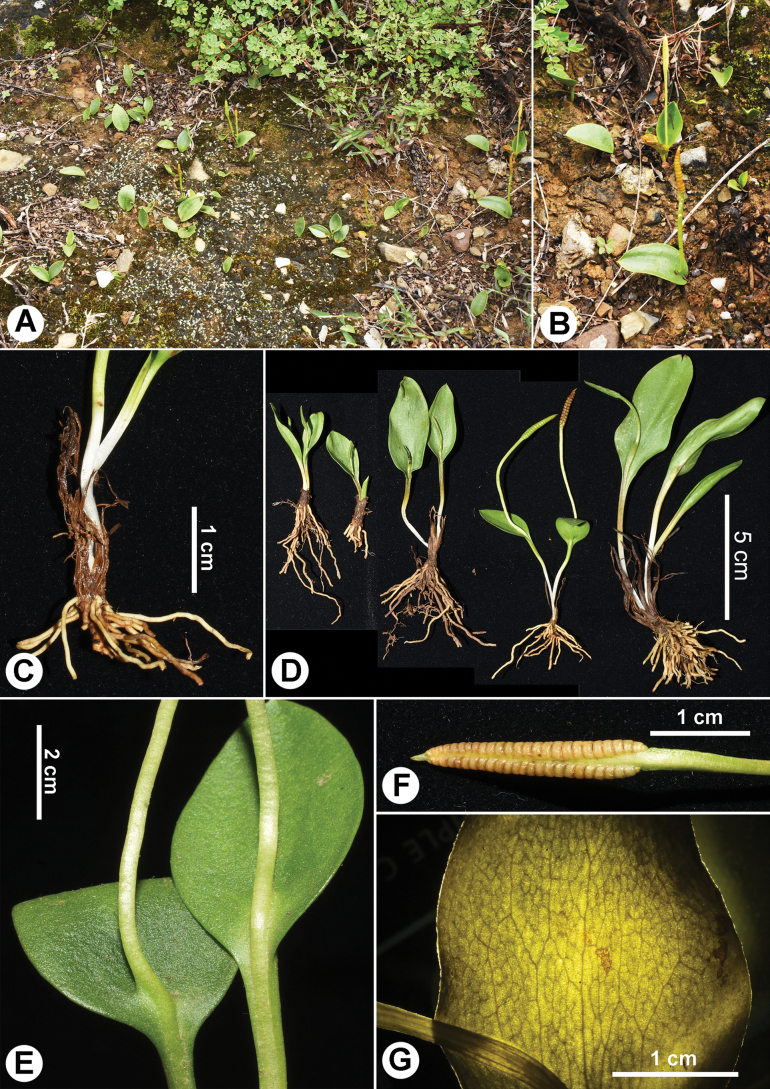
*Whittieriahengduanensis***A, B** habitat **C** lower portion of plant showing stem and roots **D** individuals in different sizes **E** trophophores and sporophore stalks **F** sporophore **G** veins showing complex-reticulate venation.

#### Diagnosis.

*Whittieriahengduanensis* is similar to *W.engelmannii* but the former has up to 25 (–50) roots per rhizome and 10–20 large areolae per trophophore, whereas the latter has fewer than 15 roots per rhizome and 0–8 areolae per trophophore.

**Figure 3.**
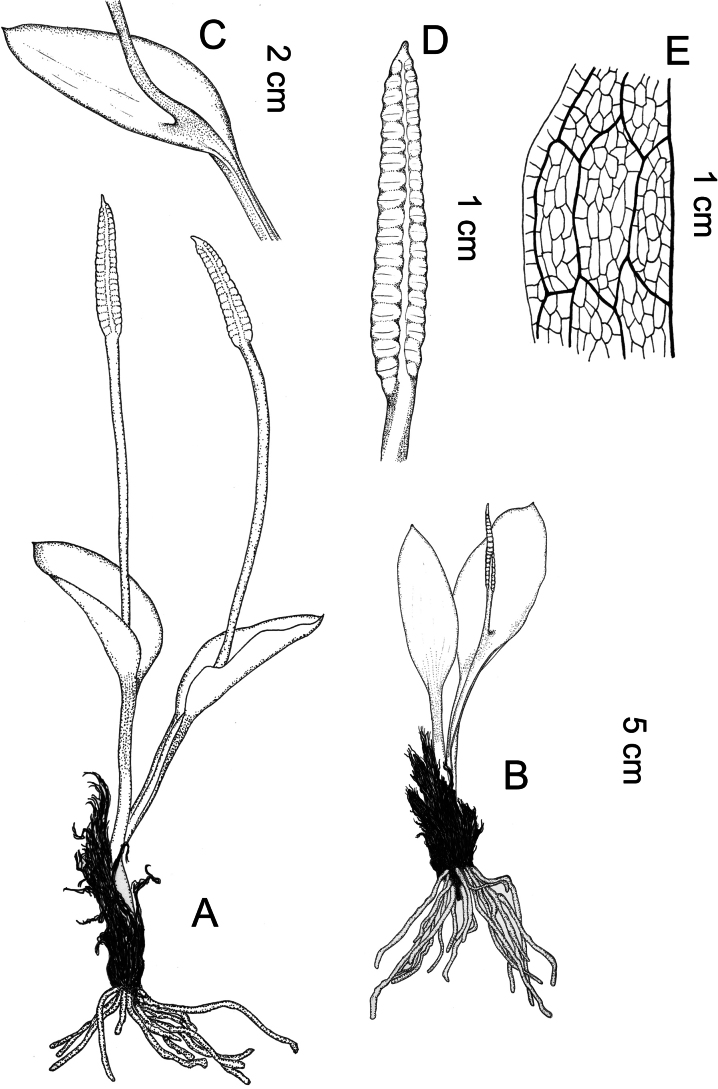
*Whittieriahengduanensis***A, B** habit **C** trophophore and base of sporophore **D** sporophore spike **E** veins showing complex-reticulate venation.

#### Description.

Plants 10–13 cm tall. Rhizomes erect, cylindrical, diam. 6–7 mm, with numerous black residual trophophore stalks. Leaves 1–2 per rhizome. Roots fleshy, up to 25 (–50) per rhizome, ca. 0.6 mm diam. Leaf stalks 4.5–7.5 cm long, 1.5–2 mm diam.; the lower part is buried in the soil, grey. Trophophores nearly circular to ovate, papery, 1.6–6 cm long, 1–3 cm broad at the middle, apex acute, narrowed toward the base. Venation complex-reticulate (also called “double venation” with small areolae inside a large areola), with included veinlets. Sporophores arising at ground level, the base is slightly attached to sporophores; stalks 3.5–7 cm long; spike 1.2–1.8 cm long, ca. 2.5 mm diam., 16–23 pairs of sporangia per spike.

#### Geographical distribution.

This species is found in western Sichuan (Batang, Daocheng, Daofu, Derong, Maerkang, Xiangcheng, Yajiang, Yuexi), northwestern Yunnan (Lijiang, etc.), and eastern Xizang (Mangkang, Zayu).

#### Habitat.

This species grows in basic soil under shrubs in dry and hot river valleys at elevations of 2500–4000 m.

#### IUCN Red List category.

Based on the field investigations, this species has a relatively wide distribution and large population sizes in southwest China and should be classified as Least Concern (LC), based on current information and following the International Union for Conservation of Nature and Natural Resources guidelines (IUCN 2024).

#### Etymology.

The species epithet *henduanensis* is based on the distribution of this species in the Hengduan Mountain.

#### Additional specimens examined.

China • **Sichuan**: Daocheng county, Aug. 23, 1937, *T.T.Yu 12889* (PE01622937); 23 Aug. 1981, *Qingzang Exp. 4234* (PE01384314) • Aug. 17, 1973, *s.c. 2519* (KUN0803070 2519) • Daofu, 18 Sept. 1934, *Harry Smith 12236* (PE00405282) • 16 Aug. 1960, *Sichuan Drug Exp. 15532* (NAS00143448) • Derong, 03 Aug. 1981, *Qingzang Exp. 3174* (PE01384316, PE01384315, HITBC053830) • Maerkang, 18 Jul. 1960, *Sichuan Drug Exp. 22092* (NAS00143451) • Yuexi, 22 Sept. 1960, *Sichuan Drug Exp. 26220* (NAS00143450) • Xiangcheng, 31 Jul. 1981, *Qingzang Exp. 3115* (PE01384313, HITBC053829) • 12 Sept. 1937, *T.T.Yu 13319* (PE00405281, PE00405283, PE00405286) • **Xizang**: Zayu, Sept. 1935, *C.W.Wang 66172* (PE00405289); Sept. 1935, *C.W.Wang 66236* (KUN0803067) • 13 Aug. 1961, *Xizang Complex Exp. 2703* (PE00405290) • 16 Aug. 1965, *Yongtian Zhang & Kaiyong Lang 1541* (KUN0803072) • **Yunnan**: Lijiang, 04 Jul. 1965, *Jinsha River Exp. 4539* (PE01593067); *T.T.Yu 13319* (KUN0803063).

## Supplementary Material

XML Treatment for Whittieria hengduanensis

## ﻿Additional information

### Author ORCIDs

Zhen-Long Liang https://orcid.org/0000-0002-9848-5055

### Data availability

All of the data that support the findings of this study are available in the main text.

## ﻿Appendix 1

Taxon sampling, voucher information, and GenBank accession numbers used in this phylogenetic analysis.

**Table A1.** A new fern species, *Whittieriahengduanensis* (Ophioglossaceae) from Sichuan, Xizang and Yunnan, China.

Species name	Voucher	Provenances	references	chloroplast complete genome	*rbcL*	*trnL* intron & *trnL-F* spacer	*rps4-trnS* spacer
*Cheiroglossapalmata*	Eric Schuettpelz 1771	Unknow			MW620387		
*Goswamiaaletum*	M. Patel MP765	India	Patel et al. 2018		MK120497		
*Goswamiacostatum*	S. Petchsri 2018-29 (BCU)	Unknow			MN524768		MN524788
*Goswamiacostatum*		India				MW666160	
*Goswamiaeliminatum*	H.K. Goswami HK004	India	Patel et al. 2018		MK120496		
*Goswamiagomezianum*	Burrows 5767 (K)	Zambia	Hauk et al. 2003		AY138419		
*Goswamiagujaratense*	K.S. Rajput et al. MIPDG023-18	India	Patil et al. 2018		MH229473		
*Goswamiahitkishorei*	M. Patel s.n. (CAL)	India	Patel and Reddy 2019		MK360156	MK358465	
*Goswamiaindicum*		India			MW666155	MW666168	
*Goswamiamalviae*	M. Patel BLAT112082 (BLAT)	India	Patel et al. 2018		MF184998		
*Goswamiareticulatum*	NL7	India			MW666154	MW666164	
*Haukiacrotalophoroides*	A. Grusz 112 (DUKE)	San Jose, Costa_Rica	Zhang et al. 2020		MN524769		
*Ophiodermapendula*		Khanh Hoa, Vietnam	Zhang et al. 2020		MN524766		MN524718
*Ophioglossumcalifornicum*		USA		KC117178			
*Ophioglossumchaloneri*	MPHKG001	Jharkhand, India	Goswami et al. 2020		MH605181	MH605183	
*Ophioglossumcoriaceum*	NSW:Papadopulos AP708	Lord Howe Island, Australia	Papadopulos et al. 2011		JF950813	JF950935	
*Ophioglossumgramineum*	RGB Kew 1981-6838 (K)	London, UK	Hauk et al. 2003		AY138412	MW666162	
*Ophioglossumkawamurae*		Japan	Shinohara et al. 2013		AB626648		
*Ophioglossumnamegatae*	TNS:764351	Ibaraki, Japan	Ebihara et al. 2010		AB574675		
*Ophioglossumnudicaule*	W. M. Chu et al. 15999	Yunnan, China	Zhang et al. 2020		MN524772		MN524792
*Ophioglossumparvifolium*	BAS10	India			MW666157	MW666167	
*Ophioglossumparvum*	R. Knapp 4647 (P, MO)	Taiwan Island	Zhang et al. 2020		MN524787		MN524805
*Ophioglossumpetiolatum*	L.B. Zhang et al. 9022	D ´a ˘ắk L ´a ˘ắk, Vietnam	Zhang et al. 2020		MN524777		MN524797
*Ophioglossumpetiolatum*	Z.R.He et al. MT-168	Xizang, China	Zhang et al. 2020		MN524774		MN524794
*Ophioglossumpusillum*	Nekola 8069 (COE)	Iowa, USA	Hauk et al. 2003		AY138413		
*Ophioglossumpycnostichum*	SCBI-SIGEO-13_0145	Unknow	Erickson et al. direct submission		KP644048		
*Ophioglossumrichardsiae*	Burrows 5756 (K)	Zambia	Hauk et al. 2003		AY138415	AY138451	
Ophioglossumthermalevar.nipponicum	TNS:1108349	Japan			AB574680		
Ophioglossumthermalevar.thermale	TNS:764007	Okinawa, Japan	Ebihara et al. 2010		AB574679		
*Ophioglossumvulgatum*	Z.L.Liang 1283	Sichuan, China	Zhang et al. 2022		OL539473	OL519781	OL519796
*Whittieriaengelmannii*	BRIT:Gostel485	Texas, USA			OL537713		
*Whittieriaengelmannii*	MEXU:1163958	Mexico			MH028789		
*Whittieriaengelmannii*	MO:G. Yatskievych 15-025	Unknow		OR777260			
*Whittieriahengduanensis*	Z.L. Liang et al. 1956	Mangkang, Xizang	this study		PQ407856	PQ407862	PQ407859
*Whittieriahengduanensis*	Z.L. Liang et al. 1958	Batang, Sichuan	this study		PQ407857	PQ407863	PQ407860
*Whittieriahengduanensis*	Z.L. Liang et al. 1959	Yajiang, Sichuan	this study		PQ407858	PQ407864	PQ407861
